# Genome editing for disease resistance in pigs and chickens

**DOI:** 10.1093/af/vfz013

**Published:** 2019-06-25

**Authors:** Chris Proudfoot, Simon Lillico, Christine Tait-Burkard

**Affiliations:** The Roslin Institute and Royal (Dick) School of Veterinary Studies, University of Edinburgh, Easter Bush, Midlothian, UK

**Keywords:** breeding, chickens, disease resistance, genome editing, pigs

**Implications**

• Genome editing technology enlarges the tool box of trait-selective breeding.
• Methods for genome editing have developed over the past decades, making the technology more efficient and specific.
• Technology to generate edited pigs and chickens is developing alongside genome editors to generate animals faster and more affordable.
• For two major pig diseases, it has been shown that resistant animals can be generated that are refractory to infection. In chickens there are promising laboratory results but no genome-edited, resistant chickens yet.
• Genome editing allows us to overcome bottlenecks in trait-selective breeding and allows the incorporation of genetic traits from other breeds, related species, or laboratory results.
• Two major hurdles still to be faced prior to the implementation of this promising technology are consumer acceptance and the regulatory framework.

## Introduction

For thousands of years, humans have used selective breeding to improve desirable traits in both livestock and companion animals. In livestock, targeted breeding has been common practice since the British Agricultural Revolution of the 18th century, with measurable production traits such as feed conversion in cattle or wool production in sheep actively selected for. In the late 20th century, genomic selection was added to the livestock breeding tool box; by reading specific locations in the genome and assigning them to measurable production traits, faster improvement in livestock production efficiency has been achieved. One of the inherently difficult production traits to measure is resistance to a specific disease, as animals with less severe symptoms or pathology may simply have been exposed to less pathogen. Experimental infections guaranteeing equal pathogen exposures are expensive and require large numbers of animals for genetic association studies, making them ethically questionable. Genome editing offers new opportunities to livestock breeding for disease resistance, allowing the direct translation of laboratory research into disease-resistant or resilient animals.

## How Are Genome-Edited Pigs and Chicken Made?

Genome editors are custom enzymes that allow scientists to cut the DNA strands in the nucleus of a cell at a specific position. The researcher can then influence how the DNA is repaired, introducing very precise genetic changes at a target locus in their species of interest. This technology has been revolutionary and provides exciting possibilities for the production of livestock resistant to viral diseases. Such opportunities are particularly pertinent given state efforts to improve global food security and reduce food waste throughout the production chain.

The most prominent editor technology today, CRISPR/Cas, uses a 20 nucleotide RNA guide to target its enzyme component to a designated locus in the genome. The probability of off-target cutting with a high fidelity Cas enzyme is very low, because with four potential base combinations at each of the 20 nucleotides there are over one trillion unique guide combinations. Once the enzyme has cut the DNA strands, the predominant repair pathway in most cells is nonhomologous end joining, an error-prone process which often introduces small insertions or deletions into the genetic code at the break site. If the target is within a gene, such perturbations can result in a disruption to the function of that gene, potentially leading to a loss of protein function. This can be very useful to basic science as it allows researchers to discover functions associated with novel genes. For many applications, a more precise change to the genome is required. To that end, scientists regularly make an alternative DNA repair process called homology-directed repair. To do this, researchers provide a novel DNA sequence alongside the CRISPR/Cas reagents, whereby the cellular repair machinery uses the new DNA as a template when fixing the break. This approach facilitates the introduction of defined changes at the genomic target locus and has sufficient refinement to alter a single nucleotide, allowing precise modification of gene function. Finally, by introducing a pair of editors, it is possible to generate two concurrent DNA breaks on the same chromosome. The cellular repair machinery then joins the ends of the cut sites, promoting the deletion of the intervening sequence. All the editor reagents introduced to the cell are rapidly degraded, with only the alteration to the genomic sequence remaining to be propagated following cell division.

**Figure 1.**
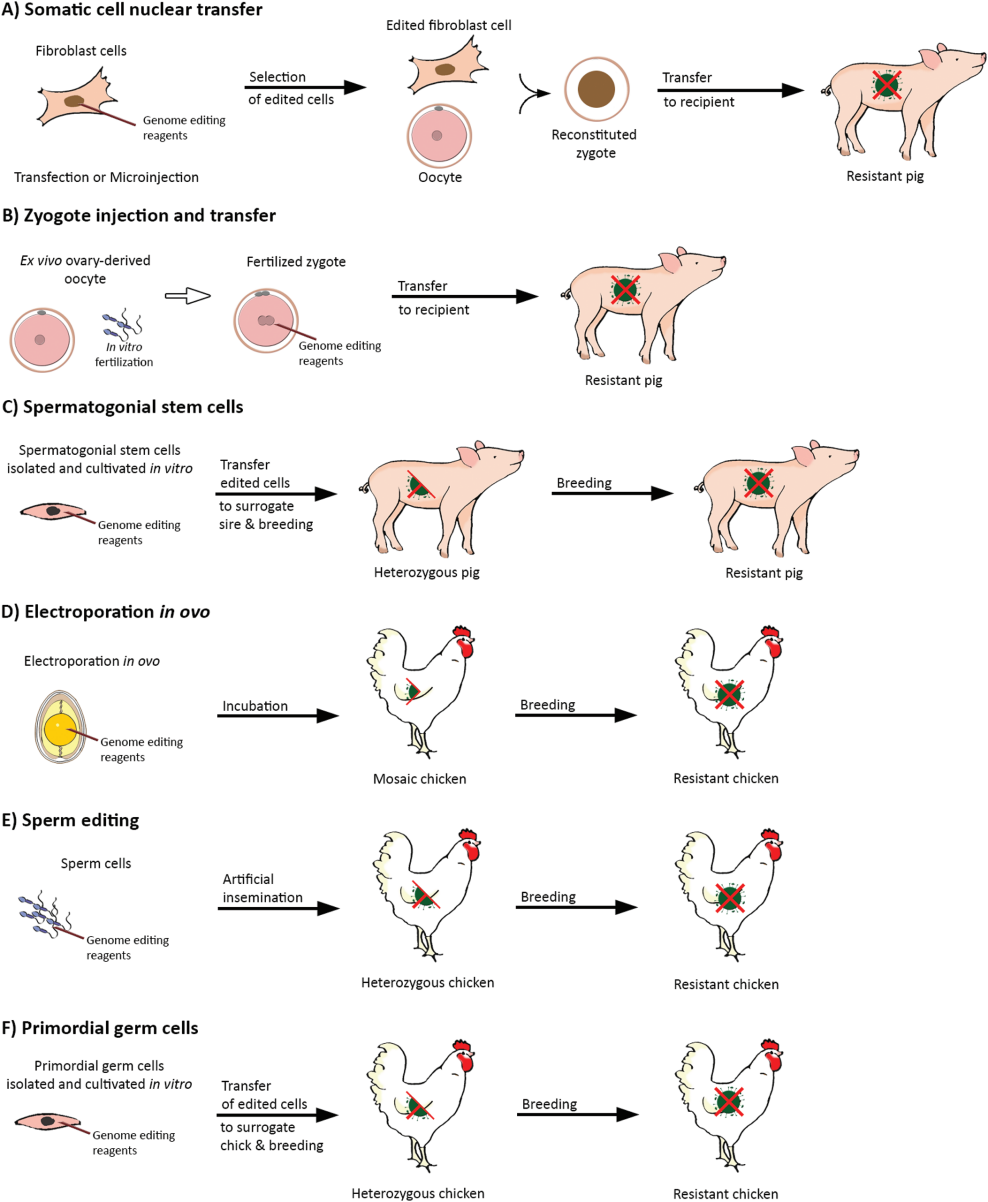
Methods to generate genome-edited pigs and chickens. (A–C) Editing in pigs. (A) Somatic cell nuclear transfer or more commonly known as cloning. Genome editing is performed in fibroblast cells, which are cultivated in vitro. A specific edit can be selected for before transfer of the nucleus into an enucleated oocyte before transfer to a recipient gilt or sow. (B) Ex vivo ovary (often slaughterhouse-derived) derived oocytes are fertilized in vitro to yield zygotes for editing or zygotes harvested from donor gilts or sows. Genome editing reagents are microinjected into the zygotes and transferred to a recipient. (C) Spermatogonial stem cells are isolated, cultivated, and edited in vitro prior to transfer to a surrogate sire. Heterozygous offspring often need to be mated before yielding a resistant pig. (D–F) Editing in chickens. (D) Electroporation in ovo. Genome editing reagents are electroporated into the embryo in ovo. Resulting chickens are often mosaic and breeding of resistant chickens is only possible if the germ cells are edited. (E) Editing of sperm by lipofection or electroporation can generate heterozygous offspring following artificial insemination. They often have to be mated to yield, homozygous chickens. (F) Primordial germ cells are isolated from embryos, cultivated, and edited in vitro. Selected edited cells are transferred to the blood stream of an embryo where they migrate to the gonad and develop into germ cells. Breeding with the resulting offspring is required to generate homozygous chickens.

Genome editing has been applied to a wide variety of agricultural species including salmonids, poultry, and ruminants. However, due to its global economic value, relatively short generation time, and multiparous nature, the most edited livestock species to date is the pig. There are two main methods widely used for the generation of edited pigs: cloning of edited fibroblasts or direct injection of the zygotes with editor reagents. Both work well, and each has specific advantages. In cloning, fibroblast cells can be maintained in the lab for prolonged periods. This allows researchers to introduce editor reagents into the cultured cells typically by lipofection, electroporation, or microinjection. Editing events in each cell of a population can be characterized and individual cells with the desired alteration to their genome selected for the cloning process, whereby the fibroblast cell is fused with an enucleated oocyte shell in a process called somatic cell nuclear transfer (Figure 1A). The reconstituted “zygote” is then transferred to a recipient gilt or sow (Carlson et al., 2012). Despite the benefit of being able to prescreen the donor cells, cloning is generally inefficient with hundreds of reconstituted zygotes being transferred to a single recipient. Cloning also yields reduced litter sizes when compared with standard breeding and offspring often demonstrate reduced viability.

As an alternative to cloning, newly fertilized zygotes can be directly microinjected with genome-editing reagents and transferred immediately back to the oviduct of a recipient animal (Figure 1B). In contrast to cloning, this approach (Lillico et al., 2013) results in the efficient establishment of pregnancies and robust litters. However, without the prescreening of cells that is routine in cloning, offspring from direct zygote manipulation inevitably encompasses a range of editing outcomes, since selection of a specific edit is not possible. Porcine zygotes can also be generated by maturation of oocytes extracted from slaughterhouse-derived ovaries and in vitro fertilization. Unfortunately, in vitro fertilization in pigs often results in polyspermy, rendering the resulting embryo inviable. However, in this controlled environment, editing rates can be increased and costs and animal use reduced.

An emerging alternative to these proven methods could be the use of surrogate sires. As a first step towards this goal, pigs have been edited to remove a gene required for male fertility, generating an empty spermatagonial stem cell niche in the testis (Park et al., 2017). Spermatogonial stem cells can be isolated and cultured in vitro, opening the possibility to edit and characterize these cells before transfer to a recipient (Park et al., 2019) (Figure 1C).

Genetic modification of poultry poses unique challenges due to the very different physiology of the avian egg compared with a mammalian oocyte. As a result, isolation and transfer of a chicken yolk is not practical. One approach that has been taken is in ovo electroporation of editing reagents, which allowed the analysis of gene function in the neural crest (Gandhi et al., 2017). However, others reported that electroporation resulted in mosaicism with editing limited to a subset of cells as the chicken embryo is already much further developed when an egg is laid compared with a zygote (Veron et al., 2015) (Figure 1D). As a result, it is unlikely that this approach could be efficiently utilized to generate edited birds. An alternative approach involves sperm transfection–assisted gene editing, whereby sperm are lipofected with editing reagents before use in artificial insemination (Cooper et al., 2017) (Figure 1E). However, advances in chicken stem cell technology show the greatest promise for genome editing in chicken. Primordial germ cells (stem cells that eventually develop into germ cells) can be isolated from the blood of developing chicks in ovo and cultured in vitro. As with mammalian fibroblasts, these cells can be edited and selected in vitro before transfer into the bloodstream of a stage-matched recipient where they migrate to and populate the developing gonad. The chicken embryo is accessed through an opening in the egg shell, which is sealed again until the chicken hatches. Genome editing in primordial germ cells has been successfully demonstrated by a number of groups (Park et al., 2014; Taylor et al., 2017; Idoko-Akoh et al., 2018) and one group has generated modified birds (Park et al., 2014). The founder birds generated from this editing method are chimeric due to the presence of preexisting germ cells. The resulting offspring generated from breeding with the founders will be a mixture of edited or nonedited. Recipient chicken embryos devoid of germ cells are currently being developed that will significantly increase the efficiency of this process (M. McGrew, unpublished results) (Figure 1F).

Genome editors will undoubtedly have a significant role on the generation of disease-resistant animals as exemplified below. It is important to note that currently the technology is limited to modifying a single gene or a SNP with large effects; however, disease resistance in many cases is likely to be a polygenic trait. Multiplexing technology is under development such that in the future polygenic traits could be altered in a single step.

## For Which Diseases Has Genome Editing Shown Progress So Far?

### Pigs

#### Porcine reproductive and respiratory syndrome virus. 

Porcine reproductive and respiratory syndrome (PRRS) is arguably the most economically important pig disease worldwide. The causative agent of PRRS is an arterivirus, named PRRS virus (PRRSV), that affects pigs of all ages but most importantly causes late-term abortions and stillbirth in sows and severe respiratory disease in piglets with severe morbidity and high mortality. PRRSV also incapacitates the pig’s immune response, providing an ideal breeding ground for severe secondary infections, mostly by bacteria, which leads to increased use of antibiotics. PRRSV exclusively infects cells of the monocyte/macrophage lineage and two macrophage-specific proteins, CD163 and CD169, were identified as receptors for the virus: CD169 acting on the surface of the cells and CD163 inside the internalizing transport vesicles (Calvert et al., 2007; Van Gorp et al., 2008). The virus was thought to attach to CD169 to be taken up into the cells; however, genome-edited pigs lacking CD169 were not resistant to PRRSV infection (Prather et al., 2013). CD163 on the other hand is thought to act through a key-lock interaction with the virus to allow it to escape from the internalizing transport vehicles into the cytosol where it replicates. CD163 consists of nine globular domains, organized like beads on a string, with domain 5 determined to mediate the key-lock interaction allowing viral entry into pig cells (Van Gorp et al., 2010). Using genome editing to generate pigs lacking CD163 Whitworth et al. showed for the first time that this approach could be used to produce livestock resistant to important viral diseases, in this case PRRS (Whitworth et al., 2016). CD163 is known to have a range of important biological functions in homeostasis, inflammation, and immune responses. As a refinement on functional knock out of the entire CD163 protein, editing reagents were designed to remove only domain 5 leaving the remainder of the protein intact. The resulting animals were completely resistant to PRRSV infection and maintained the biological functions associated with the remaining domains of CD163 (Burkard et al., 2017; Burkard et al., 2018).

#### Porcine epidemic diarrhea virus/transmissible gastroenteritis virus. 

The two coronaviruses porcine epidemic diarrhea virus (PEDV) and transmissible gastroenteritis virus (TGEV) both cause severe diarrhea in preweaned piglets and are associated with high morbidity and mortality. In vitro host–pathogen studies identified aminopeptidase N as the receptor for TGEV and a potential receptor for PEDV (Delmas et al., 1992; Li et al., 2007). The use of genome editing to generate pigs lacking aminopeptidase N successfully showed that pigs resistant to TGEV infection could be generated. However, the edited animals remained susceptible to PEDV infection (Whitworth et al., 2019). Aminopeptidase N is important for peptide digestion in the small intestine and knockout mice were shown to have delayed mammary gland development. In humans, aminopeptidase N defects are associated with different types of leukemia and lymphoma. Therefore, further investigation into the potential consequences of the absence aminopeptidase N in pigs is warranted as it may affect the overall health and/or productivity of the animals.

#### African swine fever virus. 

African swine fever virus (ASFV) causes a severe hemorrhagic disease in domestic pigs (*Sus scrofa domesticus*) and wild boars (*Sus scrofa ferus*) with high mortality in pigs of all ages. ASFV is highly contagious and can be transmitted by soft ticks of the *Ornithodoros* genus. It was identified in and contained to Africa with occasional transmission around the Strait of Gibraltar into Portugal and Spain. In 2007 an introduction of the virus into the Caucasus region showed that the virus does not solely rely on ticks for transmission in the wild, as transport of contaminated material and direct contact between animals have been shown to be major routes of disease dissemination. Since then, the virus has spread across Eastern Europe and Russia and was recently found in Western Europe and China. ASFV poses a huge risk to the pig industry worldwide and is a limiting factor to a sustainable pig industry in many parts of Africa. Interestingly, ASFV also infects wild suids, such as warthogs (*Phacocherus africanus*) and bushpigs (*Potamocherus porcus*), without causing overt disease. Such infected wild suids are thought to act as a reservoir of the virus in Africa. In the late stages of ASFV infection, a cytokine storm, i.e., an overreaction of the immune system, is observed, which is thought to strongly contribute to the lethal outcome of disease. A comparison of the warthog and domestic pig genomes identified differences in the Rel-like domain-containing protein A (RELA, also known as p65) protein, which is involved in NF-κb cytokine signaling, was thought to underlie the different responses of the related species to ASFV infection (Palgrave et al., 2011). Researchers used genome editing to convert a key region of the encoded domestic pig protein sequence to the warthog equivalent (Lillico et al., 2016). Data on susceptibility of the edited animals to ASFV infection have yet to be reported. In this instance, it is important to differentiate between disease resistance, the ability of an animal to suppress the establishment and/or development of an infection, and disease resilience, where an infected host manages to maintain an acceptable level of productivity despite challenge pressure. Should these pigs prove to be resilient to ASFV infection it is likely that their use may not be permitted in many jurisdictions, since they could act as reservoirs of infection. However, in environments where the disease is endemic use of such animals could be beneficial.

### Chicken

#### Avian leucosis virus. 

Avian leukosis virus infection results in inappetence, diarrhea, weight loss, a reduction in eggs laid, and often causes tumor formation in the chicken. The virus is divided into six subgroups, with the avian leucosis virus subgroup J (ALV-J) shown to be responsible for major disease outbreaks in China. The cellular receptor of ALV-J was identified to be the chicken sodium/hydrogen exchanger 1 protein on the cell surface. Chicken somatic cell lines have been edited to introduce changes to this gene-conferring resistance to avian leucosis virus in vitro (Lee et al., 2017). Despite cells showing resistance to ALV-J infection, no edited chickens have been produced to date. In both mice and humans, a lack of the sodium/hydrogen exchanger 1 protein is associated with severe neurological disease; however, targeted changes to single amino acids may retain biological functions of the protein in chicken while resulting in disease resistance.

#### Avian influenza virus. 

In chickens, disease resistance to avian influenza is at the top of the wish list due to the serious impact on chicken health but also the risk of transmission to humans. Similarly, influenza A is also one of the diseases on the resistance wish list for pigs, as they can act as an intermediate host-aiding virus adaptation to humans. The acidic leucine-rich nuclear phosphoprotein-32A (ANP32A) was found to play a key role in avian influenza virus replication in both chicken and water fowl. Although the virus polymerase protein readily interacts with the avian ANP32A, the human version of the same protein supports only limited replication of the viral genome. It has been demonstrated in vitro that deletion of a small region of chicken ANP32A can prevent replication of avian influenza virus (Long et al., 2016; Long et al., 2019). Although the functional consequence of edited ANP32A has yet to be demonstrated in vivo, such approaches offer exciting opportunities that have the potential to benefit both industry and animal welfare.

As exemplified above, currently many gene editing approaches focus on targeting host genes involved in mediating entry of the virus, with a special focus on receptors. However, as the example for avian influenza shows, host genes play an important role in other steps of the pathogen replication cycle and also provide editing targets for disease resilience or resistance. More in-depth host–pathogen interaction studies, including genome-wide editing studies in vitro, will no doubt produce a variety of further candidate genes for genetic disease resistance.

An alternative antipathogen approach pursued for decades is the generation of transgenic livestock, expressing antiviral or antibacterial agents, such as enzymes or small interfering RNAs. Genome editing can be used to improve the integration efficiency of these transgenes at specific locations in the genome; however, the discussion of transgenic disease-resistant animals is beyond the scope of this review.

### How does genome editing fit within existing selective breeding structures and how will it be regulated?

**Figure 2.**
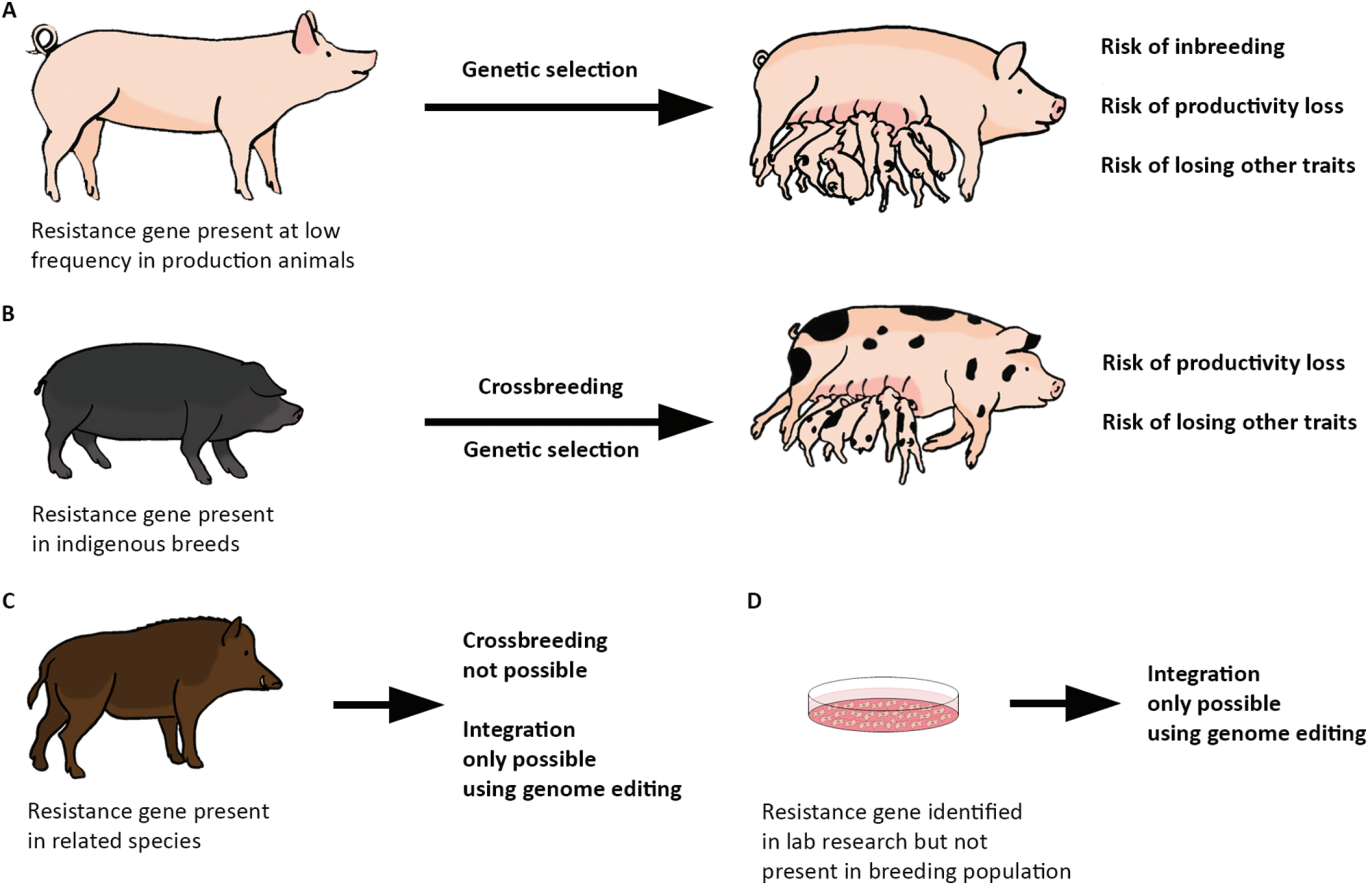
Genetic resistance to disease and how genome editing can help integrate traits into highly productive lines. (A) Genetic resistance to disease may be present in a small percentage of production animals and genetic selection for these animals may be associated with the risk of inbreeding, productivity loss, or the risk of losing other desirable traits. Genome editing allows integration of the disease-resistance trait into a wider selection of pigs, ensuring genetic variability and maintenance of desirable traits. (B) Genetic resistance to disease may be present in an indigenous or less productive breed. Crossbreeding would result in productivity loss and the risk of losing other desirable traits, such as fur color. Genome editing allows for incorporation of genetic disease resistance into highly bred lines without losing productivity. (C) Genetic resistance may be observed in a closely related species, e.g., wild boar or wild suids in the case of the domestic pig. Integration into highly bred domestic pig lines would only be possible by genome editing. (D) Resistance genes may be identified in laboratory research but not in highly bred lines, making integration into those productive animals only possible using genome editing.

Selective breeding has generated highly productive, robust animals that are adapted to a modern production environment. Livestock production is dynamic, with evolving challenges such as climate change and disease outbreaks coupled with societal pressure to reduce antimicrobial use. Selective breeding for disease resistance has proven difficult, as outbreaks are often sporadic and resistant/resilient animals often difficult to identify. In circumstances where a genetic trait for disease resistance can be identified in the breeding population, then selection through the selective breeding can be achieved. A good example of this is pigs with resistance to F18 type enterotoxigenic *E. coli*. Association studies revealed that a polymorphism in the fucosyl transferase 1 gene conferred resistance to these bacteria. There was initial concern that selection for the locus harboring this gene may counterselect for another gene associated with stress resistance. However, this proved not to be the case and genetic selection for the favorable fucosyl transferase 1 allele has been integrated into many pig-breeding programs (Coddens et al., 2008). This was possible, in part, because the favorable allele was present at sufficient prevalence (in most studies between 5% and 10%) in the breeding population to allow for selection while avoiding inbreeding. In circumstances where an allelic variant associated with a resistant phenotype is present at a much lower frequency, it may prove difficult to incorporate effective selection into a standard breeding regime without the risk of inbreeding and related longer-term productivity loss (Figure 2A). Genome editing has the potential to contribute in such circumstances, allowing the direct introgression of a beneficial allele into the offspring of diverse, highly productive animals. Similarly, disease-resistance traits associated with less productive indigenous breeds are unlikely to be introduced to highly productive populations by standard crossbreeding as this would result in a significant set-back in productivity, abrogating decades or even centuries of advances made through genetic selection (Figure 2B). In circumstances where resistance or resilience is observed in a related species, crossbreeding is simply not possible. Genome editing could bridge these gaps. One example of this is resilience of wild suids to African swine fever virus while domestic pigs can suffer from severe disease. It is not possible to crossbreed these species, so introduction of the genetics underlying resilience is not possible by this route. Genetic comparison can be used to identify the functional differences underlying such traits, and genome editing employed to introduce appropriate variants into domestic pigs (Figure 2C). Finally, with a good understanding of host–pathogen interactions, novel genetics that has not been observed in live animals can be created and tested for efficacy in a laboratory environment. This was the case for both the CD163/PRRSV and APN/TGEV examples in pigs and would be the case for the ANP32A/ influenza and the ALV-J resistance in chicken, described above. In such circumstances, integration through genome editing presents a practical route to benefit from the findings (Figure 2D). It is imperative that in such circumstances thorough phenotypic characterization of the edited animals be carried out as deletion of all or part of a functional protein could result in a loss of (systemic) biological function. A second measure worthy of consideration before embarking on an editing project is whether the gene is located within a locus that has been actively selected in breeding programs. This could indicate whether a potential target is associated with known production traits. This approach has been taken for PRRSV-resistant pigs, with evaluation as to whether the CD163 gene locus has been selected for in pig breeding programs (Johnsson et al., 2018).

## Conclusions

Overall, genome editing holds vast promise for the future production of animals resistant or resilient to disease, improving productivity and animal welfare while reducing food waste throughout the production chain. Through reduction of primary and secondary infections, it should also be possible to reduce antimicrobial use in livestock production. Technical improvements in the generation of genome editing animals will undoubtedly reduce the cost implications of this technology. The two major hurdles still to be faced prior to implementation of this promising technology are consumer acceptance and the regulatory framework. Approval of edited animals for human consumption relies on national and multinational legislation, which is currently at early stages. Encouragingly, some international jurisdictions such as Argentina and Brazil have already ruled that modifications, such as the PRRSV-resistant pig, that do not have any new genetic information integrated into the animal, will be exempt from regulation.

## About the Authors

**Dr. Chris Proudfoot** is research fellow at The Roslin Institute/University of Edinburgh since 2011. His work centers on generation of genome-modified livestock, with particular emphasis on genome editors, to improve disease resistance or to accurately model human disease. Dr. Proudfoot has worked extensively with ZFNs, TALENs, and CRISPR/Cas9 to produce a variety of edited animals. He was a member of the team that produced the first edited livestock using this method.

**Dr. Simon Lillico** is a research associate at The Roslin Institute/University of Edinburgh. He joined the institute on an industrial collaboration to produce high-value therapeutic proteins in hens eggs and then applied his expertise in lentiviral transgenesis to generate livestock models of human diseases. The rapid expansion of the field of genome editors over the last 7 yr has made practicable genome modifications which had previously been unattainable. Dr. Lillico has been at the forefront of application of these editors to livestock, creating either disease-resistant/resilient strains, or accurate models of human disease.

**Dr. Christine Tait-Burkard** is an assistant professor at The Roslin Institute/University of Edinburgh since 2017 in the departments of Genetics and Genomics and Infection and Immunity. Her research focuses on understanding host–pathogen interactions on a cellular and genetic level, developing new in vitro tools for virus research, improving and developing easy-to-use diagnostics, and devising strategies to combat viral disease in livestock in general and pigs in particular. She employs genome editing and genetic selection to generate animals genetically resistant to viral disease.
